# Rare Abdominal Wall Metastasis following Curative Resection of Gastric Cancer: What Can Be Learned from the Use of Percutaneous Catheters?

**DOI:** 10.1155/2020/3738798

**Published:** 2020-05-09

**Authors:** Arthur A. Parsee, Kerry L. Thomas, Masoumeh Ghayouri, Rutika Mehta, Kujtim Latifi, Jennifer Sweeney, Daniel Jeong, Abraham Ahmed

**Affiliations:** ^1^Department of Radiology, Moffitt Cancer Center, Tampa, Florida, USA; ^2^Department of Pathology, Moffitt Cancer Center, Tampa, Florida, USA; ^3^Department of Gastrointestinal Oncology, Moffitt Cancer Center, Tampa, Florida, USA; ^4^Department of Radiation Oncology, Moffitt Cancer Center, Tampa, Florida, USA

## Abstract

In cancer care, tissue seeding after curative resections is a known potential complication, despite precautions taken during surgical treatment. We present an uncommon case of an abdominal wall metastasis along the tract of a surgical drain following gastrectomy for gastric adenocarcinoma. To our knowledge, this is the first case of such an occurrence in the setting of a negative staging peritoneal lavage. Aside from the rarity of such a recurrence, this instance highlights an opportunity to reevaluate best practices with regard to the extent of coverage of postoperative salvage radiotherapy. The oncologic patient provides many challenges and may require multiple catheters for drainage and at times infusion of nutrition or therapeutic agents. These foreign bodies should be scrutinized both clinically and radiographically, as they may create vulnerabilities in keeping malignant diseases contained and controlled. We provide a review of the literature with reasonable treatment options for the benefit of future patients.

## 1. Introduction

Cancer treatment is multidisciplinary and comprehensive with the ultimate goal of tumor eradication to maximize survival and minimize morbidity. Complications must be handled expediently to mitigate morbidity and improve prognosis. Metastatic seeding following resection is a rare but significant occurrence warranting further therapy. Spread patterns of gastric cancer rely primarily on histology, with generic adenocarcinoma mostly involving the liver (48%), followed by the peritoneum (32%) and lung (15%), and least often to the bone (12%) [1]. The signet ring subtype of adenocarcinoma has a predilection for the peritoneum, with a lesser burden in the liver and lungs. The second consideration is tumor location, with cases divided into the cardia and noncardia categories, with the latter also demonstrating a tendency for peritoneal carcinomatosis [1]. This case highlights the presentation and timing with subsequent treatment of an abdominal wall metastasis following total gastrectomy.

## 2. Case Presentation

**Figure 1:**
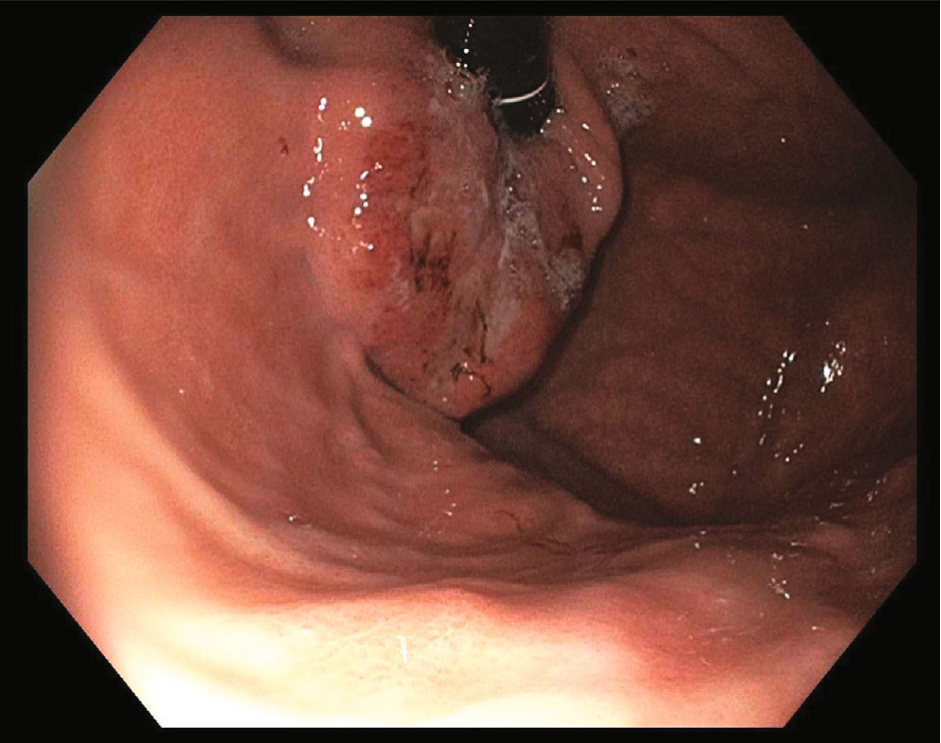
EGD photograph—an ulcerated mass 40 cm from incisors is shown, with stigmata of recent bleeding involving the gastric cardia, extending to the gastroesophageal junction.

A 62 year-old female with no significant past medical history was found to have a normocytic anemia (Hgb 8.6) on routine bloodwork. The primary care provider's workup included upper endoscopy (Figure 1) which revealed a mass 40 cm from the incisors, arising from the gastric cardia, with extension to the gastroesophageal junction. Endoscopic biopsy returned invasive moderately-differentiated gastric adenocarcinoma. Risk factors include smoking (19 pack-years; quit more than 10 years ago) and occasional alcohol consumption of 2-3 drinks per week. No complaints of dysphagia, weight loss, or change in bowel habits. Intermittent gastroesophageal reflux disease (GERD) was treated with pantoprazole. No personal or family history of cancer was noted.

**Figure 2:**
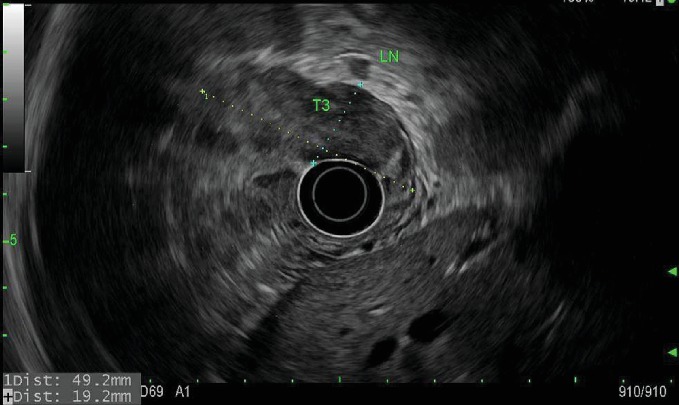
EUS—a hypoechoic mass involving two-thirds of the gastric circumference measured 4.8 × 1.9 cm, with serosal invasion and two malignant-appearing lymph nodes (one shown in field-of-view) in the paracardial region (level 16), T3N1MX by endosonographic criteria.

Staging endoscopic ultrasound (Figure 2) demonstrated a well-circumscribed hypoechoic mass measuring up to 4.8 cm with sonographic evidence of serosal invasion. Two malignant-appearing lymph nodes were identified in the paracardial region (level 16) 5 mm from the tumor, lending to staging of T3N1MX. Initial 18-fluoro-deoxyglucose (^18^FDG) positron-emission tomography/computed tomography (PET/CT) showed concentrated uptake within only the gastric mass, with a standardized uptake value (SUV) of 10.4, but no evidence of distant metastasis. Initial carcinoembryonic antigen (CEA) level was 5.6 ng/mL (normal is less than 5.2).

**Figure 3:**
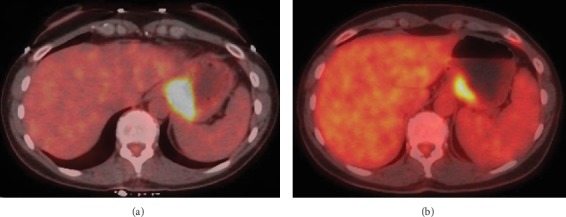
Initial axial fused PET/CT image (a) showing robust hypermetabolism of the gastric cardia (max SUV 10.4) with subsequent axial fused PET/CT image (b.) following neoadjuvant therapy with reduced size and uptake (SUV 4.3), consistent with 60% reduction in FDG concentration.

Diagnostic laparoscopy showed no peritoneal disease, and lavage washings were negative for malignant cells. A multidisciplinary approach concluded the best path towards lessening disease burden prior to a curative resection for a Siewert III gastroesophageal junction cancer [2]. Neoadjuvant chemotherapy was initiated, and it consisted of 6 cycles of 5-fluorouracil, leucovorin, oxaliplatin, and docetaxel (FLOT) with docetaxel being excluded from the last round. A restaging PET/CT (Figure 3) showed reduction in both tumor bulk and FDG avidity by approximately 60%, with CEA level decreased to 2.3 (initially 5.6).

Following standard preoperative cardiac clearance, the patient underwent a robotic-assisted total gastrectomy and D2 lymphadenectomy. En bloc resection included 4 cm of the distal esophagus as well as total omentectomy. Just prior to this, a small lymph node in the splenic hilum was sent for frozen section, which was negative for malignancy. Before construction of the esophagojejunostomy, frozen sections of the resection margins returned, which were also clear of any tumor. A Jackson-Pratt (JP) drain was placed behind the anastomosis. Endoscopic testing was negative for any leaks. A feeding jejunostomy was also placed.

**Figure 4:**
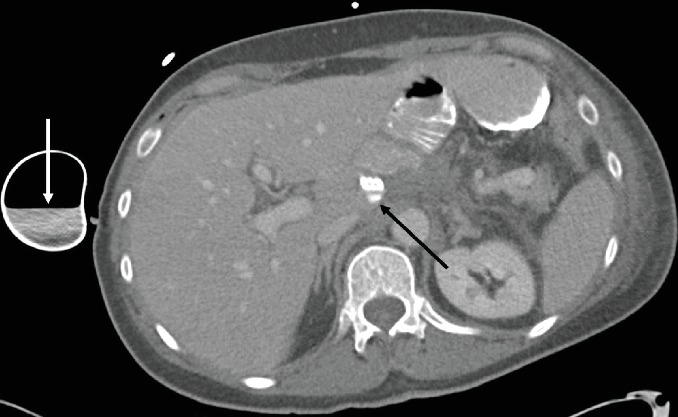
Axial postcontrast CT performed in the postoperative period showed leaked contrast concentrated at the tip of the surgical drain (black arrow). Contrast level is also present within Jackson-Pratt drain reservoir (white arrow).

Postoperative recovery was initially as expected, with routine fluoroscopic interrogation of the anastomosis showing no leak with either water-soluble or barium contrast and with normal esophageal motility. On day 4, an episode of fever, tachycardia, and hypertension occurred. Antipyretic and empiric antibiotics were started, and a CT was performed for further investigation (Figure 4). This revealed a small collection of extraluminal contrast near the tip of the surgical drain and a concentration of contrast within the drain's reservoir, both suggesting a small previously occult anastomotic leak. Endoscopy confirmed a small ulceration at the anastomosis, 35 cm from the incisors, which was effectively covered by a 7.0 × 2.3 cm EndoMAXX stent (Merit Medical, South Jordan UT, USA) traversing 31 to 38 cm from the incisors. On day 9, a repeat CT showed a new subphrenic collection as well as more leaked contrast within Morison's pouch. A CT-guided drain was placed to effectively evacuate the subphrenic collection. The remainder of the postoperative course was relatively uneventful, with the jejunostomy removed once the patient was tolerating a soft mechanical diet.

**Figure 5:**
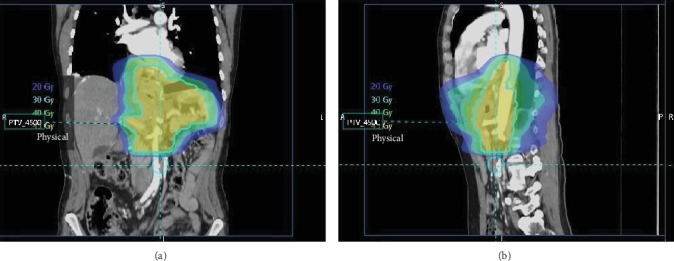
Coronal (a) and sagittal (b) overlays with isodense lines of planned external beam radiotherapy (XRT) with coverage of the surgical bed.

Surgical pathology showed both lymphovascular and perineural invasion, with tumor within 1 mm of the omental margin. Only 2 of 27 nodes were positive, without treatment effect of the primary tumor present, yielding a final stage of ypT4aN1. A regimen of salvage chemoradiation was initiated, which included capectabine, as well as 1.8 Gy fractions divided over 25 sessions, for a total dose of 45 Gy focused on the surgical bed (Figure 5).

**Figure 6:**
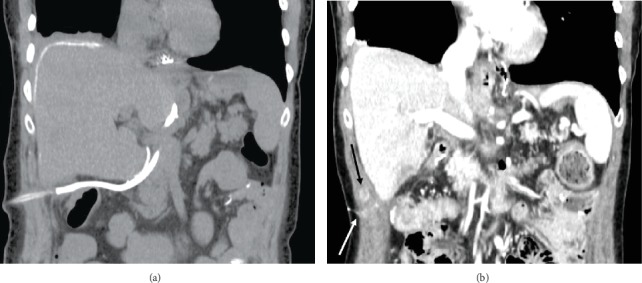
Coronal noncontrast (a) and postcontrast (b) CT reconstructions demonstrating a new vascular nodule (black arrow) in close proximity to the drain tract with the residual barium demarcating tract (white arrow).

**Figure 7:**
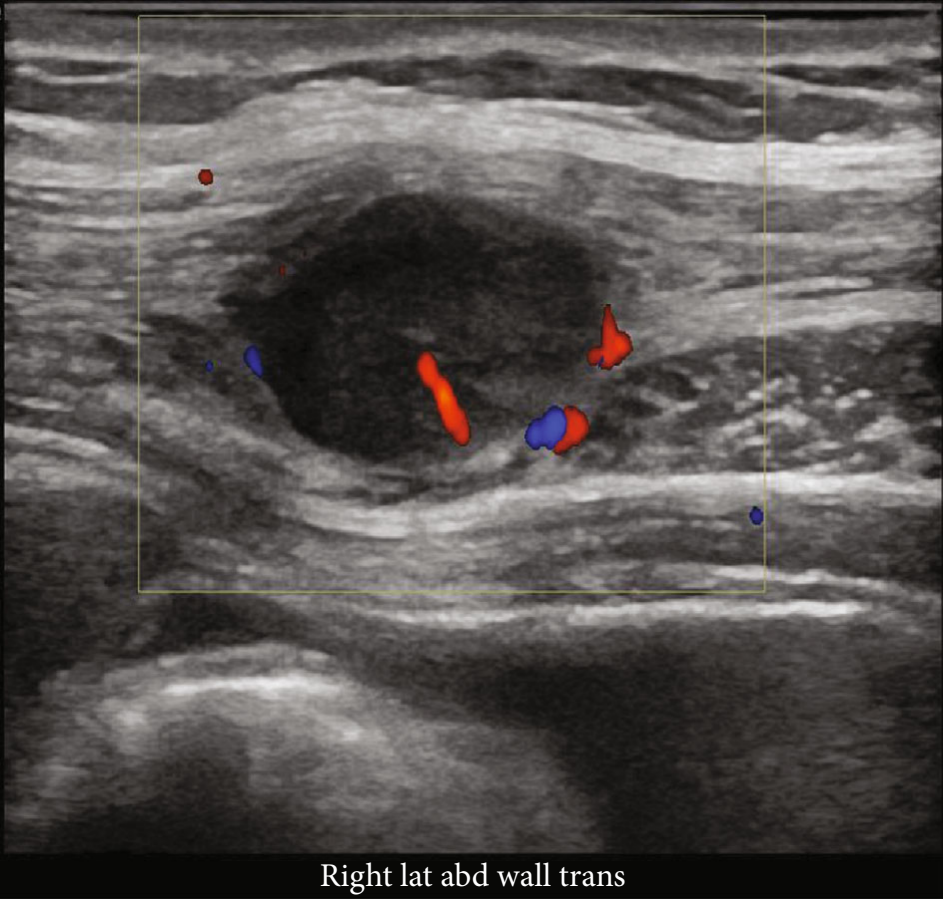
Doppler ultrasound confirming the subcutaneous nodule with internal vascularity and distortion of associated subcutaneous tissue planes, performed concurrent with image-guided biopsy.

**Figure 8:**
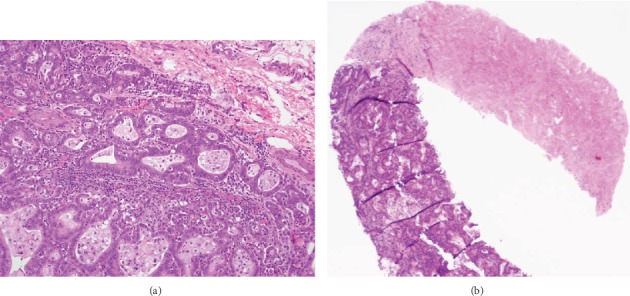
Hematoxylin and eosin-stained histology slides of primary gastric adenocarcinoma at 4x magnification, moderately-differentiated with solid nests and glandular architecture (a). Identical pattern found after biopsy of the abdominal wall mass (b) at 1x magnification.

Two months later, a near-tripling in CEA (6.4 from 2.3) was concurrent with a new CT finding of a 1.1 cm hypervascular lesion embedded within the right abdominal wall subcutaneous fat (Figure 6) in close proximity to the previous JP surgical drain. This was palpable on the physical exam. An ultrasound was performed (Figure 7) to facilitate targeted biopsy. Pathology from core biopsy (Figure 8) showed an identical histological pattern as the original gastric adenocarcinoma. Immunohistochemical analysis was performed in hopes of eliciting additional treatment options. This revealed 3+ expression of the HER2/neu receptor. HER2 testing was not performed on the original endoscopic biopsy or the gastrectomy specimen. A regimen was undertaken consisting of four cycles of calcium folinate, 5-fluorouracil, oxaliplatin, and leucovorin (FOLFOX) as well as Herceptin (trastuzumab). After treatment, the nodule was no longer palpable and had resolved on imaging. The patient is now on maintenance Herceptin alone and is disease free.

## 3. Discussion

Gastric cancer specifically is known to spread primarily via the peritoneum; the two primary paths being stomata-like orifices along the omentum and transvessel migration facilitated by hypoxic-induced factor-1*α* [3]. Esophagectomy studies have suggested a mechanical disruption of lymphatic channels as being another method for the spread [4]. These studies suggest that the peritoneal fluid at some point becomes contaminated with viable tumor cells, which can be confirmed with lavage of both the peritoneal cavity and surgical wounds. In cases of en bloc resection, a study found that wound washings were positive in 13% of instances, with drained fluid being positive in 9%, in a pool of 184 patients [5]. Studies of upper gastrointestinal cancers with anastomotic leaks are sparse; however, analogous reviews of colorectal cancers did not find that leaks had a significant impact on survival, tract or peritoneal seeding, or local recurrence [6, 7].

Peritoneal lavage has been established as an adjunct in staging, albeit of debated utility [8, 9]. A patient series with exclusive gastrointestinal malignancies found that staging lavage was only helpful in 1.3% of cases in providing useful prognostic information [10]. The most consistent risk factor to consider is the size of the primary tumor at initial staging [11]. The precise moment of seeding may be difficult to determine, as various interventions create a multitude of seeding vulnerabilities. Percutaneous biopsies, whether core or fine needle, provide one possibility of dissemination [12]. Enterostomy tubes for either drainage or feeding deliver yet another path for tumor propagation [13]. The most corroborated risk is transhepatic drainage for malignant biliary obstructions [14–16]. The prevalence of catheter tract seeding is somewhat disputed, with two reviews displaying rates ranging from as low as 6% to as high as 22% [17, 18]. In addition, soft tissue spread is not unique to GI malignancies; for example, an intracranial glioma has been reported to disperse into the peritoneum via a ventricular shunt [19]. Furthermore, seeding is not specific to the peritoneum or abdomen. Malignant effusions spreading from the pleura to the chest wall have been reported to be as high as 22% [20].

As it has been established that tract seeding can arise with a variety of tissue types and through variable media, it is reasonable to undertake protective measures to prevent tract seeding. While surgical literature has described the importance of resecting open biopsy tracts, this appears to be specific to sarcomas and not necessarily applicable to needle biopsies [21]. An argument has been made to justify prophylactic radiation to chest tube sites in the setting of mesothelioma, in the form of a single-dose 10 Gy fraction [22]. While there are several treatment options that may be applied to GI malignancies, it is generally acknowledged that isolated tract metastases, once removed or resolved, generally do not recur [23] as currently holds true in this case.

This report serves as both an example and warning that gastrointestinal cancers with anastomotic leaks can seed catheter tracts, even with a negative staging lavage of the peritoneum. Any catheter site must be scrutinized, as it will extend beyond the irradiated field. Tumor markers and imaging are helpful in diagnosing abdominal wall metastases.

Informed consent was obtained and a copy is available upon request. The case was anonymized, with the exemption from IRB approval.

## Data Availability

Not applicable.
